# An siRNA library screen identifies CYLD and USP34 as deubiquitinases that regulate GPCR-p38 MAPK signaling and distinct inflammatory responses

**DOI:** 10.1016/j.jbc.2023.105370

**Published:** 2023-10-20

**Authors:** Norton Cheng, JoAnn Trejo

**Affiliations:** 1 Department of Pharmacology, School of Medicine, University of California, San Diego, La Jolla, California, USA; 2 Biomedical Sciences Graduate Program, School of Medicine, University of California, San Diego, La Jolla, California, USA

**Keywords:** inflammation, PAR1, p38 MAPK, thrombin, ubiquitin

## Abstract

G protein–coupled receptors (GPCRs) are highly druggable and implicated in numerous diseases, including vascular inflammation. GPCR signals are transduced from the plasma membrane as well as from endosomes and controlled by posttranslational modifications. The thrombin-activated GPCR protease-activated receptor-1 is modified by ubiquitin. Ubiquitination of protease-activated receptor-1 drives recruitment of transforming growth factor-β–activated kinase-1–binding protein 2 (TAB2) and coassociation of TAB1 on endosomes, which triggers p38 mitogen-activated protein kinase–dependent inflammatory responses in endothelial cells. Other endothelial GPCRs also induce p38 activation *via* a noncanonical TAB1-TAB2–dependent pathway. However, the regulatory processes that control GPCR ubiquitin–driven p38 inflammatory signaling remains poorly understood. We discovered mechanisms that turn on GPCR ubiquitin–dependent p38 signaling, however, the mechanisms that turn off the pathway are not known. We hypothesize that deubiquitination is an important step in regulating ubiquitin-driven p38 signaling. To identify specific deubiquitinating enzymes (DUBs) that control GPCR-p38 mitogen-activated protein kinase signaling, we conducted a siRNA library screen targeting 96 DUBs in endothelial cells and HeLa cells. We identified nine DUBs and validated the function two DUBs including cylindromatosis and ubiquitin-specific protease-34 that specifically regulate thrombin-induced p38 phosphorylation. Depletion of cylindromatosis expression by siRNA enhanced thrombin-stimulated p38 signaling, endothelial barrier permeability, and increased interleukin-6 cytokine expression. Conversely, siRNA knockdown of ubiquitin-specific protease-34 expression decreased thrombin-promoted interleukin-6 expression and had no effect on thrombin-induced endothelial barrier permeability. These studies suggest that specific DUBs distinctly regulate GPCR-induced p38–mediated inflammatory responses.

G protein–coupled receptors (GPCRs) are a large family of cell surface receptors with broad functions in physiology and disease and the target of 34% of approved drugs (1, 2). Recent advances in GPCR structure and pharmacology have been significant. However, an aspect of GPCR regulation that remains poorly understood is the role of posttranslational modifications. Phosphorylation is well studied and critical for controlling GPCR signaling and trafficking *via* recruitment of the multifunctional β-arrestin-1 and 2 adaptor proteins (3). In addition to phosphorylation, ubiquitination has been implicated in regulation of GPCR signaling and trafficking (3). Like phosphorylation, ubiquitination is typically inducible and a reversible process. However, there is a limited understanding of how ubiquitination controls GPCR signaling.

Protein ubiquitination is best known to target proteins for degradation *via* the 26S proteasome and lysosome (4). Ubiquitin is covalently linked to lysine residues of substrate proteins through the sequential action of E1, E2, and E3 ubiquitin-modifying enzymes. While proteins are predicted to be modified with ubiquitin at least once during their lifetime, ubiquitination has been studied only for a small subset of approximately 40 GPCRs (3, 5, 6). In most studies, ubiquitin has been linked to GPCR endosomal-lysosomal trafficking or to the proteasome for degradation (7, 8, 9). However, emerging studies indicate that for certain GPCRs, ubiquitin promotes direct interaction with signaling effectors (10). Thus, the function of ubiquitination may vary depending on the GPCR, cell type, and physiological function.

Several studies have examined the role of ubiquitin in propagating GPCR signaling from the plasma membrane (11, 12). We showed that agonist stimulation of a subset of endothelial GPCRs promotes ubiquitin-dependent p38 mitogen-activated protein kinase (MAPK) activation on endosomes *via* a noncanonical pathway (10, 13, 14). Activation of the GPCR protease-activated receptor-1 (PAR1) by thrombin induces ubiquitin-dependent recruitment of transforming growth factor-β–activated protein kinase-1–binding protein-2 (TAB2), which associates with TAB1 (10, 14, 15). TAB1 binds directly to the p38α isoform inducing a conformational change resulting in autophosphorylation and activation (16, 17). The ubiquitin-driven PAR1 signaling is specific to p38 MAPK, as thrombin activation of extracellular signal–regulated kinase-1/2 (ERK1/2) proceeds through the canonical three-tiered kinase cascade (10). Like PAR1, the purinergic P2Y1 receptor also promotes ubiquitin-driven p38 activation through a TAB1-TAB2–dependent pathway (10, 13), indicating that this pathway is used by multiple GPCRs. Indeed, other GPCR agonists including histamine (H_1_ or H_2_ receptors) and prostaglandin E_2_ (EP_4_ prostanoid receptor) activate p38α through a noncanonical TAB1–dependent pathway in endothelial cells (13).

Ubiquitination is a reversible process and deubiquitination is important for governing ubiquitin-dependent cellular responses such as endocytic trafficking and cell signaling. The human genome encodes approximately 100 deubiquitinating enzymes (DUBs). DUBs serve multiple functions including removal of ubiquitin from protein substrates, which can rescue proteins from degradation or modulate signaling, ubiquitin chain editing, and recycling of ubiquitin, which ensures that ubiquitin re-enters the ubiquitin cellular pool (18, 19). Although ubiquitination is important for regulating GPCR signaling and trafficking, the role of DUBs in controlling GPCR signaling and the impact on biological functions remains poorly understood. In this study, we employed an RNA interference library screen in two cell types to identify DUBs that specifically regulate thrombin-stimulated p38 MAPK signaling and endothelial inflammatory responses.

## Results

### An siRNA library screen identifies a subset of DUBs that regulate GPCR p38 MAPK signaling

**Figure 1:**
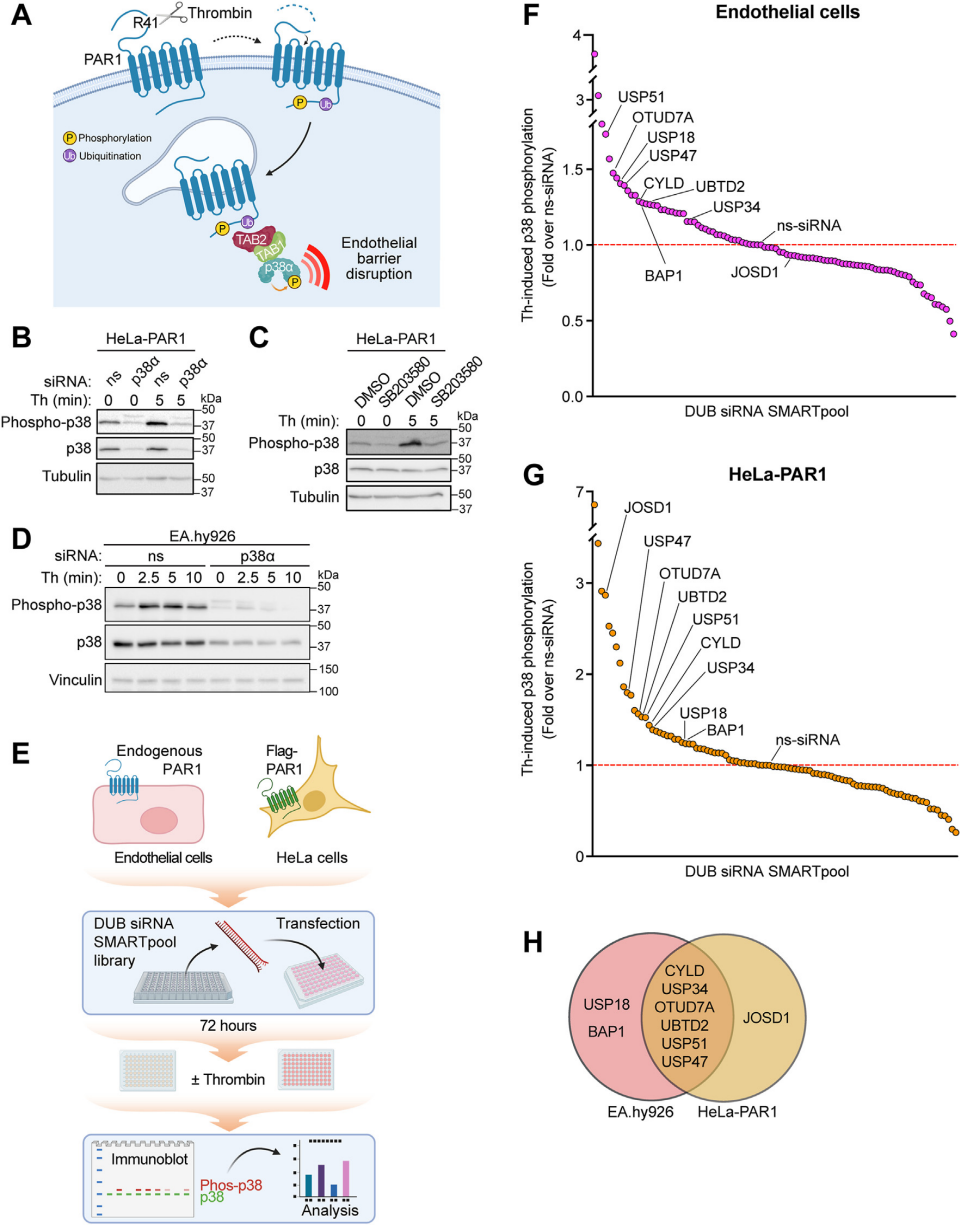
**An siRNA library screen identifies nine DUBs that regulate thrombin-stimulated p38 signaling.***A*, thrombin cleaves and activates PAR1 at an N-terminus arginine (R) 41 site. After activation, PAR1 is phosphorylated and ubiquitinated, ubiquitin drives TAB1-TAB2–dependent p38 activation from endosomes and disruption of endothelial barrier. *B* and *D*, HeLa and endothelial EA.hy926 cells transfected with either p38α-specific or nonspecific (ns) siRNA were stimulated with thrombin (Th) and p38 phosphorylation detected by immunoblot (n = 1). *C*, HeLa cells pretreated with p38 inhibitor SB203580 or dimethylsulfoxide vehicle control were stimulated with thrombin and p38 phosphorylation determined (n = 1). *E*, workflow diagram. Endothelial EA.hy926 cells and HeLa cells transfected with Human DUB siRNA SMARTpool library and ns siRNA control were stimulated with thrombin for either 7.5 min (endothelial) or 5 min (HeLa), performed in duplicate. Phosphorylation of p38 was detected by immunoblot and quantified (n = 1) for the two different cell types. *F* and *G*, endothelial and HeLa cell siRNA library screen data are expressed as the fold change in p38 phosphorylation normalized to total p38 expression relative to ns siRNA control (*red dotted line*). *H*, Venn diagram includes DUBs known to cleave K63-linked ubiquitin, which resulted in upregulated thrombin-stimulated p38 phosphorylation in both endothelial and HeLa cell lines. DUB, deubiquitinating enzyme; PAR1, protease-activated receptor-1; TAB, transforming growth factor-β–activated kinase-1–binding protein.

Thrombin activation of PAR1 triggers ubiquitin-driven p38 MAPK inflammatory signaling *via* a noncanonical TAB1-TAB2–dependent pathway on endosomes to promote endothelial barrier disruption (10, 13, 20) (Fig. 1*A*). Although mechanisms that turn on ubiquitin-driven GPCR-p38 signaling have been identified (14), the mechanisms that turn off noncanonical p38 signaling are not known. We hypothesize that deubiquitination is a key step in terminating ubiquitin-dependent p38 signaling induced by GPCRs. To identify DUBs that specifically regulate thrombin-stimulated p38 MAPK signaling, an siRNA library screen targeting 96 different mammalian DUBs was conducted in two different cell types including human umbilical vein endothelial cells (HUVEC)-derived EA.hy926 cells and HeLa cells. Endothelial EA.hy926 cells express endogenous PAR1 (21, 22), whereas PAR1 is stably expressed in HeLa cells (23). A siRNA specifically targeting the p38α MAPK isoform reduced p38 expression and blocked thrombin-stimulated p38 phosphorylation detected with a pan anti-phospho-p38 antibody in HeLa cells and endothelial cells (Fig. 1, *B* and *D*), indicating that the p38α isoform is a major effector of thrombin signaling (20, 24). A similar inhibitory effect on thrombin-induced p38 activation was observed with SB203580, an inhibitor of the p38α and β isoforms, consistent with autoactivation as shown in our previous studies (10, 13) (Fig. 1, *B* and *C*). These data indicate that thrombin induces p38α isoform signaling *via* autoactivation in endothelial cells and HeLa cells (10, 13, 20, 24).

The DUB siRNA library screen workflow performed in endothelial and HeLa cells transfected with nonspecific siRNA and 96 different SMARTpool siRNAs targeting human DUBs is illustrated in Figure 1*E*. After transfection, cells were left untreated or treated with thrombin at a single time point and the induction of p38 phosphorylation was measured by immunoblotting, quantified, and analyzed (Fig. 1*E*). Nine siRNA SMARTpools targeting different DUBs known to cleave mono- or K63-linked ubiquitin markedly increased thrombin-induced p38 phosphorylation in endothelial and HeLa cells compared to nonspecific siRNA control cells (Fig. 1, *F* and *G*), suggesting that a subset of DUBs control thrombin-stimulated p38 signaling. Six of the DUBs including cylindromatosis (CYLD), ubiquitin-specific protease (USP)34, USP51, USP47, OTU deubiquitinase 7A (OTUD7A), and ubiquitin domain containing 2 (UBTD2) regulated thrombin-p38 signaling in both cell types, whereas USP18- and BRCA1-associated protein 1 (BAP1) were effective in endothelial cells and Josephin domain containing 1 (JOSD1) in HeLa-PAR1 cells (Fig. 1*H*). Data from the complete SMARTpool siRNA DUB library screen performed in endothelial cells and HeLa cells are shown in Figs. S1 and S2 and Datasets S1 and S2. To our knowledge, this study is the first comprehensive siRNA library screen of all mammalian DUBs conducted to identify modulators of GPCR signaling.

### A subset of DUBs regulate thrombin-stimulated p38 MAPK signaling over time

**Figure 2:**
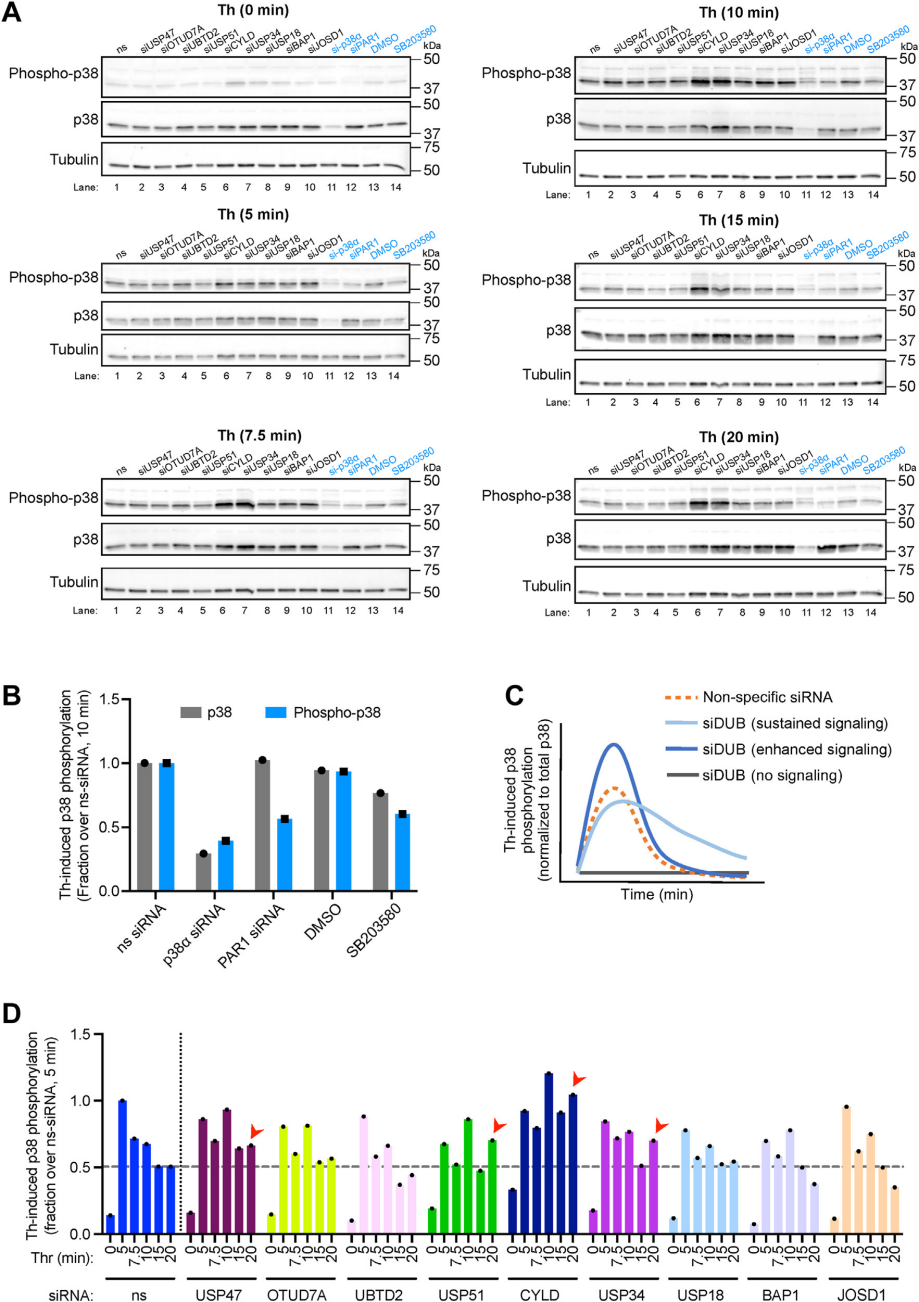
**A subset of DUBs regulate thrombin-stimulated p38 MAPK signaling over time.***A*, endothelial EA.hy926 cells transfected with either non-specific (ns) siRNA, nine DUB SMARTpool siRNA, or p38α- or PAR1-specific siRNAs were serum-starved and stimulated with thrombin (Th) for the indicated times. Non-transfected endothelial cells were pre-treated with dimethylsulfoxide or the p38 inhibitor SB203580 and then stimulated with thrombin. Cell lysates were immunoblotted as indicated and quantified (n = 1). *B*, changes in thrombin-induced p38 phosphorylation in ns-, p38α-, PAR1-siRNA transfected cells or dimethylsulfoxide, SB203580 treated cells are shown as a fraction of ns siRNA control (n = 1). *C*, graphical illustration of anticipated impact of DUB depletion on p38 signaling over time. *D*, changes in thrombin-stimulated p38 phosphorylation in DUB depleted endothelial cells are expressed as a fraction of ns siRNA at 0 min over time (n = 1). *Red arrowhead* shows markedly elevated thrombin-stimulated p38 phosphorylation compared to ns siRNA control cells. DUB, deubiquitinating enzyme.

DUBs that specifically regulate thrombin-activated p38 MAPK inflammatory signaling have not been identified. To verify the specificity of SMARTpool siRNAs targeting the candidate DUBs at modulating thrombin-stimulated p38 signaling, a time course analysis in endothelial cells was performed. Endothelial cells were transfected with nonspecific siRNA or SMARTpool siRNA targeting the nine candidate DUBs and then stimulated with or without thrombin over a 20 min time course. PAR1- and p38α-specific siRNA and the SB203580 inhibitor were used as controls. Thrombin induced a marked increase in p38 phosphorylation detectable as early as 5 min that remained elevated at 7.5 and 10 min in nonspecific siRNA transfected cells (Fig. 2, lane 1, *B* and *D*). However, siRNA-mediated depletion of p38α or PAR1 expression markedly reduced thrombin-stimulated p38 phosphorylation at various times compared to nonspecific control siRNA, (Fig. 2, *A* lanes 1, 11, and 12 and *B*). A similar effect was observed in endothelial cells pretreated with the p38 inhibitor SB203580 (Fig. 2, *A* lanes 1, 13, and 14 and *B*). We predicted that depletion of a critical DUB by siRNA may modulate PAR1-induced p38 signaling through loss of function exhibited as no signaling or gain of function displayed as either increased signaling or sustained signaling compared to nonspecific siRNA as illustrated in Figure 2*C*. Analysis of the effect of DUB depletion by SMARTpool siRNA on thrombin-p38 signaling showed that loss of USP47, USP51, CYLD, and USP34 expression resulted in a marked sustained increase in p38 signaling compared to nonspecific siRNA (Fig. 2, A lanes 1, 2, 5–7 and *D*). In addition, siRNA-mediated loss of CYLD expression increased the magnitude of thrombin-induced p38 activation compared to control nonspecific siRNA transfected cells (Fig. 2, *A* lanes 1, and 6 and *D*). However, SMARTpool siRNA mediated depletion of OTUD7A, UBTD2, USP18, BAP1, and JOSD1 failed to alter thrombin-stimulated p38 signaling compared to siRNA control cells over time (Fig. 2A lanes 1, 3, 4, 8–10). These data indicate that four different DUBs may function to regulate thrombin-induced p38 signaling.

### CYLD, USP34, USP47, and USP51 expression in endothelial and HeLa cells

**Figure 3:**
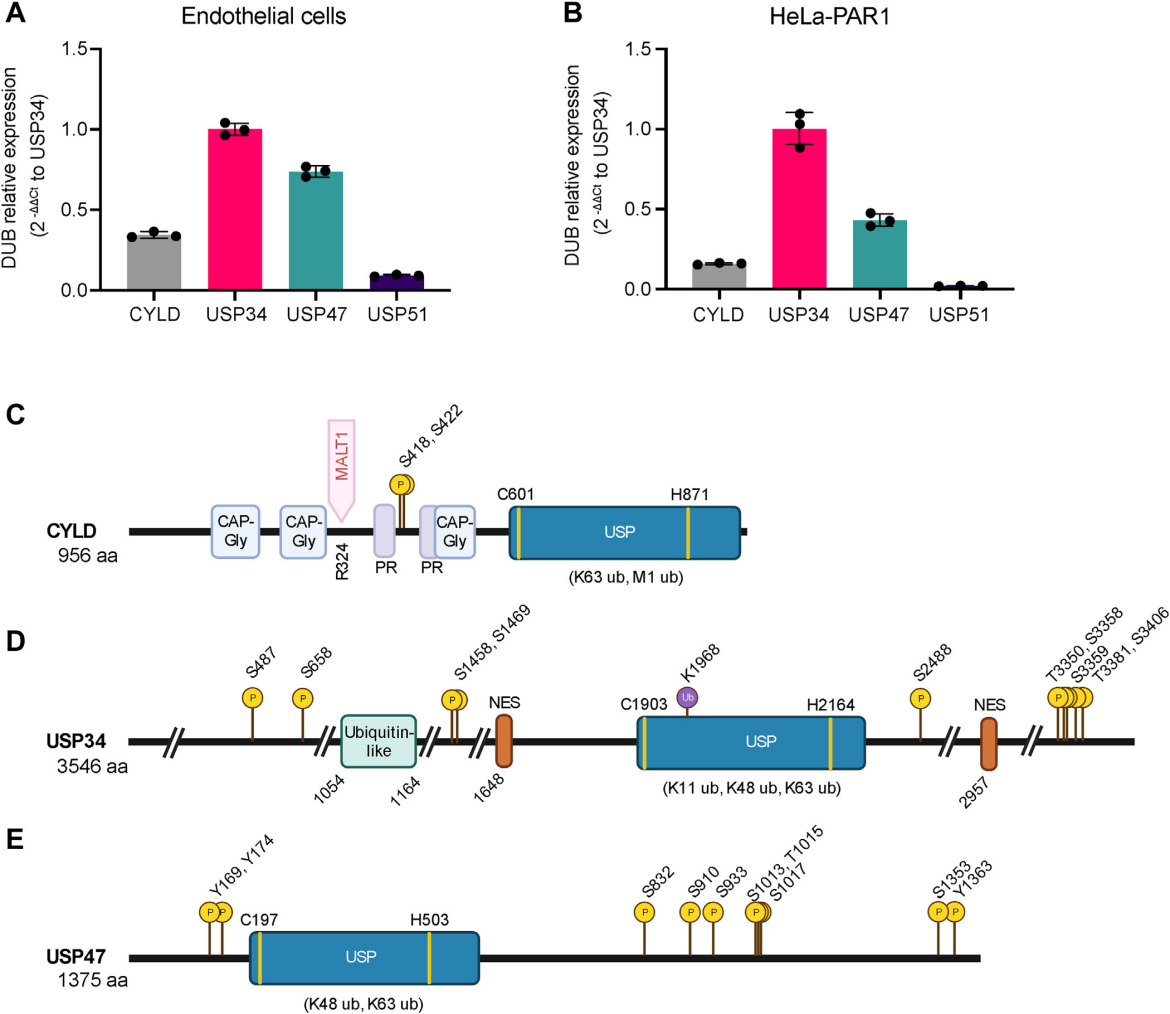
**DUB mRNA expression, protein domains and phosphorylation sites**. CYLD, USP34, USP47 and USP51 expression was determined in endothelial EA.hy926 cells (*A*) and PAR1-expressing HeLa cells (*B*) by real time RT-qPCR. Data (mean ± S.D, n = 3) normalized to 18S rRNA are expressed as (2^-ΔΔCt^) relative to USP34 expression. CYLD (*C*), USP34 (*D*) and USP47 (*E*) cartoon illustrations of domains, phosphorylation, and ubiquitination sites. CYLD contains three cytoskeleton-associated protein-glycine-rich domain (CAP-Gly), two proline rich (PR) regions and ubiquitin-specific protease domain (USP). CYLD is cleaved by MALT1. USP34 contains a ubiquitin-like domain, USP domain and two nuclear export signals (NES). USP47 contains a USP domain. DUBs contain multiple sites of phosphorylation and USP34 possesses one ubiquitination site identified in PhosphositePlus. DUB, deubiquitinating enzyme; RT-qPCR, quantitative real-time reverse transcription PCR.

To interrogate the function of candidate DUBs implicated in thrombin-induced p38 signaling, we first examined the expression of endogenous CYLD, USP34, USP47, and USP51 in endothelial cells and HeLa cells quantitatively using quantitative real-time reverse transcription PCR (RT-qPCR). USP34 exhibited the highest expression in endothelial cells and HeLa cells compared to the other DUBs (Fig. 3, *A* and *B*). Considerable expression of USP47 and CYLD were also detected in both cell types (Fig. 3, *A* and *B*). However, USP51 displayed the lowest expression compared to the other DUBs and was barely detectable in HeLa cells (Fig. 3, *A* and *B*) and therefore eliminated from further analysis.

CYLD, USP34, and USP47 belong to the cysteine protease class of DUBs that utilize an active site cysteine (C) residue as part of a catalytic triad containing an adjacent histidine (H) and a third acidic residue within the USP domain to facilitate hydrolysis of ubiquitin linkages (Fig. 3, *C*–*E*). CYLD has been highly studied and contains two N-terminal cytoskeleton–associated protein glycine-rich domains, two proline-rich motifs, a mucosa-associated lymphoid tissue lymphoma translocation protein-1 (MALT1) cleavage site at arginine (R)-324 and a C-terminal USP domain that mediates preferential cleavage of K63- and methionine-1–linked ubiquitin (Fig. 3*C*). In contrast to CYLD, USP34 is a poorly characterized DUB that contains an N-terminal ubiquitin-like domain, two nuclear export signals and a C-terminal USP domain that cleaves K11-, K48-, and K63-linked ubiquitin (Fig. 3*D*) (25). USP47 contains a N-terminal USP domain that cleaves K48- and K63-linked ubiquitin (Fig. 3*E*), like USP34.

DUB activity is controlled by relative abundance, protein–protein interaction and subcellular localization (26, 27). DUBs are also regulated by posttranslational modifications. Numerous phosphorylation sites have been identified in CYLD, USP34, and USP47 in discovery mass spectrometry studies reported on PhosphoSitePlus (28). The sites of phosphorylation with greater than ten reports are shown for CYLD, USP34, and USP47 (Fig. 3, *C*–*E*). CYLD phosphorylation serine (S)418 and S422 sites are well documented in independent studies (29, 30) and shown in Figure 3*C*. USP34 phosphorylation S718 and S1751 sites have been identified in HeLa cells (31) and are not shown in Figure 3*D*, since there were less than ten reports, whereas no additional USP47 phosphorylation sites have been identified. Intriguingly, only USP34 ubiquitination at a single site K1968 has been reported on PhosphoSitePlus and detected in twelve discovery mass spectrometry studies (Fig. 3*D*).

**Figure 4:**
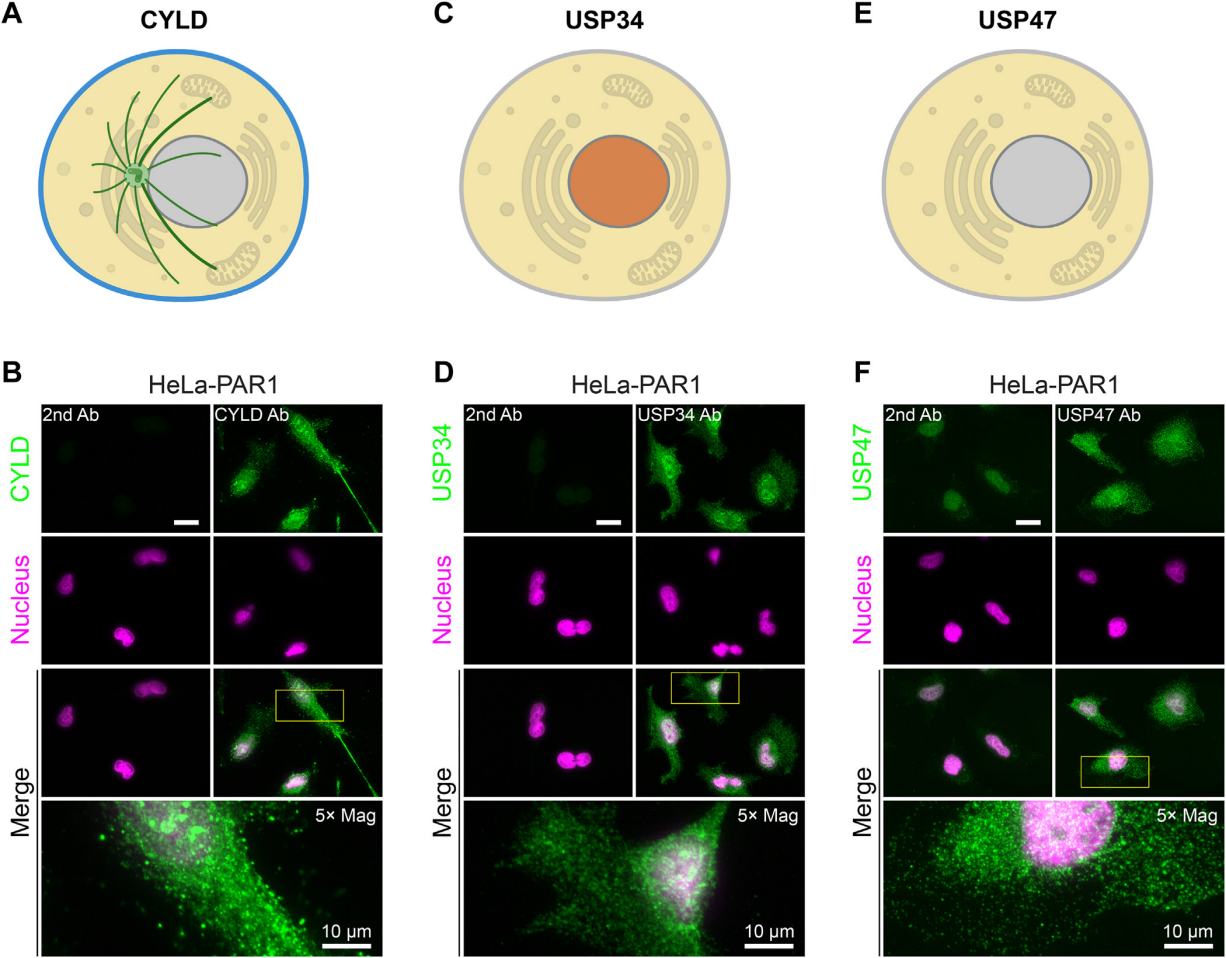
**DUB candidate subcellular localization.** Cartoon illustrations of CYLD (*A*), USP34 (*C*), and USP47 (*E*) subcellular localization at the plasma membrane (*blue*), cytoskeleton (*green*), cytoplasm (*yellow*), and nucleus (*orange*). *Gray* indicates no localization. *B*, *D*, and *F*, HeLa cells were fixed, processed, immunostained for endogenous CYLD, USP34 or USP47, stained with Hoechst to detect the nucleus, and imaged by confocal microscopy. The scale bar represents 20 μm. Insets are 5× magnifications with scale bar = 10 μm. CYLD, cylindromatosis; DUB, deubiquitinating enzyme; USP, ubiquitin-specific protease.

Next, we examined the subcellular localization of the candidate DUBs, including CYLD, USP34, and USP47. In UniProt (32), CYLD has been reported to reside in several subcellular compartments depending on the cell type, including the plasma membrane, cytoplasm in HEK293 cells (33) and cytoskeleton in ciliated epithelial cells (34) (Fig. 4*A*). In HeLa cells, endogenous CYLD localized to the cytoplasm with some detection in the nucleus (Fig. 4*B*). USP34 is reported to reside in the cytoplasm and nucleus in UniProt (Fig. 4*C*) and shown to regulate the cytoplasmic Axin protein and Wnt/β-catenin signaling (35). USP34 also reported to localize to the nucleus in HeLa cells (36) (Fig. 4*C*). Consistent with these studies, we found that USP34 resides in the cytoplasm and nucleus in HeLa cells (Fig. 4*D*). USP47 has been shown to localize mainly in the cytoplasm in HeLa cells (37) (Fig. 4*E*). We also detected USP47 predominantly in the cytoplasm (Fig. 4*E*). These findings suggest that multiple regulatory processes including posttranslational modifications, protein–protein interactions, and subcellular localization exist to control DUB activity.

### Depletion of CYLD, USP34, and USP47 by single siRNAs alter thrombin-induced p38 MAPK signaling in endothelial cells

**Figure 5:**
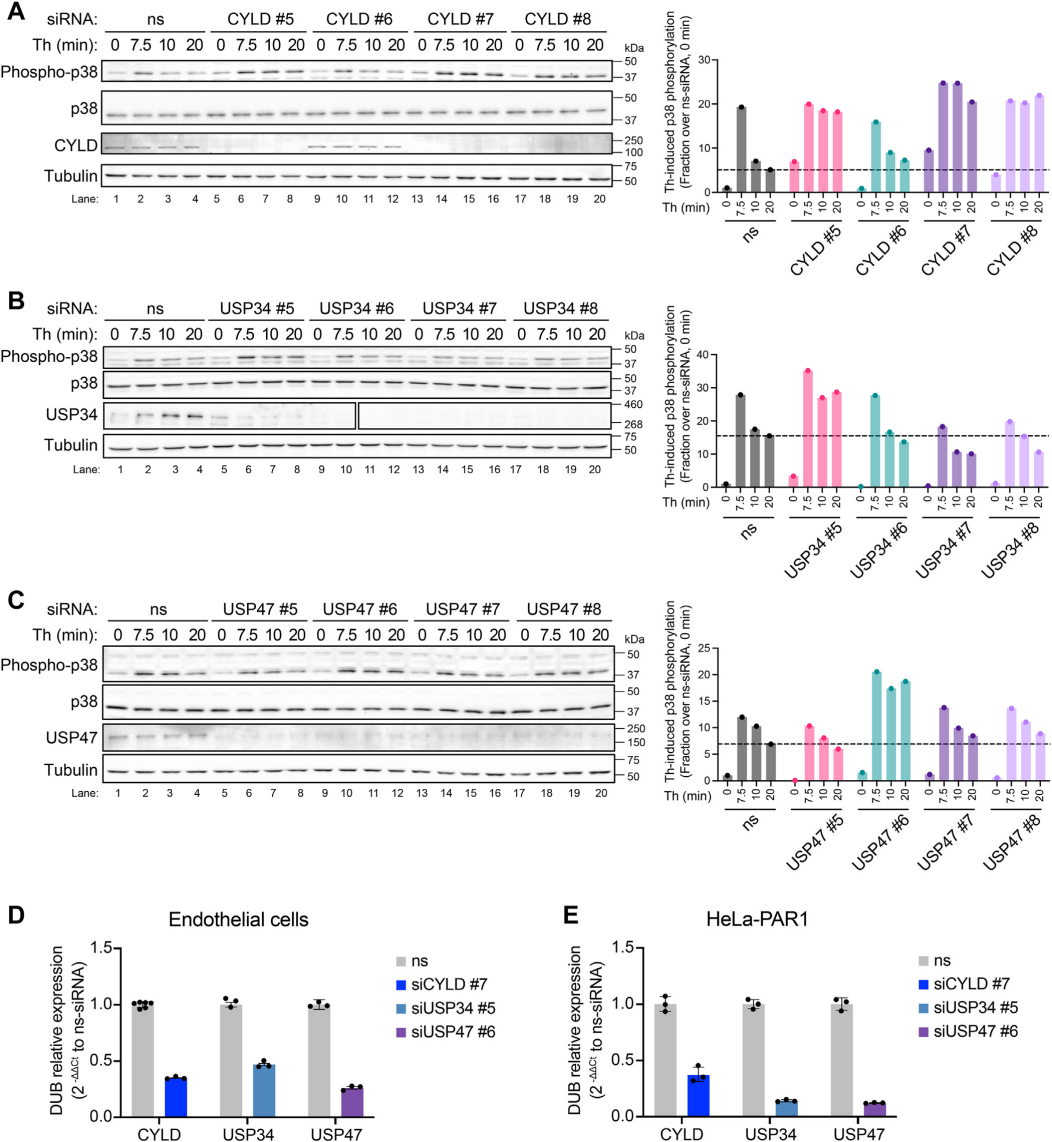
**Depletion of CYLD, USP34, and USP47 by individual SMARTpool siRNAs alter thrombin-induced p38 phosphorylation.** Endothelial EA.hy926 cells were separately transfected with four individual SMARTpool siRNAs for CYLD (*A*), USP34 (*B*), and USP47 (*C*) or nonspecific (ns) siRNA control. Cells were then treated with or without thrombin (Th) for the indicated times. Cell lysates were immunoblotted as shown. Changes in thrombin-induced p38 phosphorylation were quantitated (n = 1) and the data are expressed as the fraction of p38 phosphorylation over ns siRNA at 0 min. The effect of selected individual DUB siRNAs on CYLD, USP34, and USP47 mRNA degradation was determined by RT-qPCR in endothelial EA.hy926 cells (*D*) and HeLa cells (*E*). The data (mean ± S.D., n = 3) normalized to 18S rRNA are expressed as 2^-ΔΔCt^ relative to ns siRNA. CYLD, cylindromatosis; DUB, deubiquitinating enzyme; RT-qPCR, quantitative real-time reverse transcription PCR; USP, ubiquitin-specific protease.

To examine the specificity of SMARTpool siRNAs, we separately examined the effect of the four individual siRNAs constituting the SMARTpools on thrombin-stimulated p38 phosphorylation over time. In nonspecific siRNA transfected endothelial cells, thrombin promoted a marked increase in p38 phosphorylation at 7.5 min that decreased by 10 min and returned to basal levels by 20 min (Fig. 5*A* lanes 1–4). Similarly, a substantial increase in p38 activation peaked at 7.5 min, following thrombin stimulation in endothelial cells transfected with all three of the four CYLD siRNAs (Fig. 5*A* lanes 5-–0). However, in cells transfected with CYLD siRNA #5, #7, and #8 thrombin-stimulated p38 signaling was enhanced and sustained compared to nonspecific siRNA control (Fig. 5*A* lanes 1-8 and 13-20). The CYLD #5, #7, and #8 siRNAs effectively depleted cells of endogenous CYLD protein expression detected by immunoblot compared to control cells (Fig. 5*A* lanes 1–8 and 13–20). However, siRNA #6 failed to reduce CYLD expression and as expected thrombin-p38 signaling was comparable to control siRNA transfected cells (Fig. 5*A* lanes 9–12 *versus* 1–4). Moreover, depletion of CYLD with siRNAs #5, #7, #8 appeared to modestly enhance basal p38 phosphorylation compared to siRNA control cells (Fig. 5*A* lanes 1 *versus* 5, 13, and 17). Unlike CYLD, none of the four individual siRNAs targeting either USP34 or USP47 affected basal p38 phosphorylation in endothelial cells (Fig. 5, *B* and *C*). However, USP34 siRNA #5 and USP47 siRNA #6 were found to alter thrombin-p38 signaling. Cells transfected with USP34 siRNA #5 showed a marked and sustained increase in p38 phosphorylation, following thrombin stimulation compared to control cells (Fig. 5*B* lanes 1–8) and a considerable loss of endogenous USP34 expression detected by immunoblot (Fig. 5*B* lanes 1–8). Similarly, thrombin enhanced p38 phosphorylation in endothelial cells transfected with USP47 siRNA #6, which also showed diminished USP47 expression by immunoblot compared to nonspecific siRNA-treated control cells (Fig. 5*C* lanes 1–4 and 9–12). The individual siRNAs including CYLD #7, USP34 #5, and USP47 #6 were also validated by examining their capacity to specifically knockdown gene expression in endothelial and HeLa cells using RT-qPCR. Consistent with the loss of DUB expression detected by immunoblot, endothelial cells transfected with the individual DUB-specific siRNAs showed substantial decrease in CYLD, USP34, and USP47 mRNA expression compared to nonspecific siRNA control (Fig. 5*D*). The individual DUB specific siRNAs caused a similar marked reduction in CYLD, USP34, and USP47 mRNA expression in HeLa cells (Fig. 5*E*). These data suggest that CYLD, USP34, and USP47 may function as key regulators of thrombin-stimulated p38 MAPK signaling.

### Depletion of CYLD or USP34 with multiple individual siRNAs significantly increased thrombin-induced p38 phosphorylation but not ERK1/2 phosphorylation

**Figure 6:**
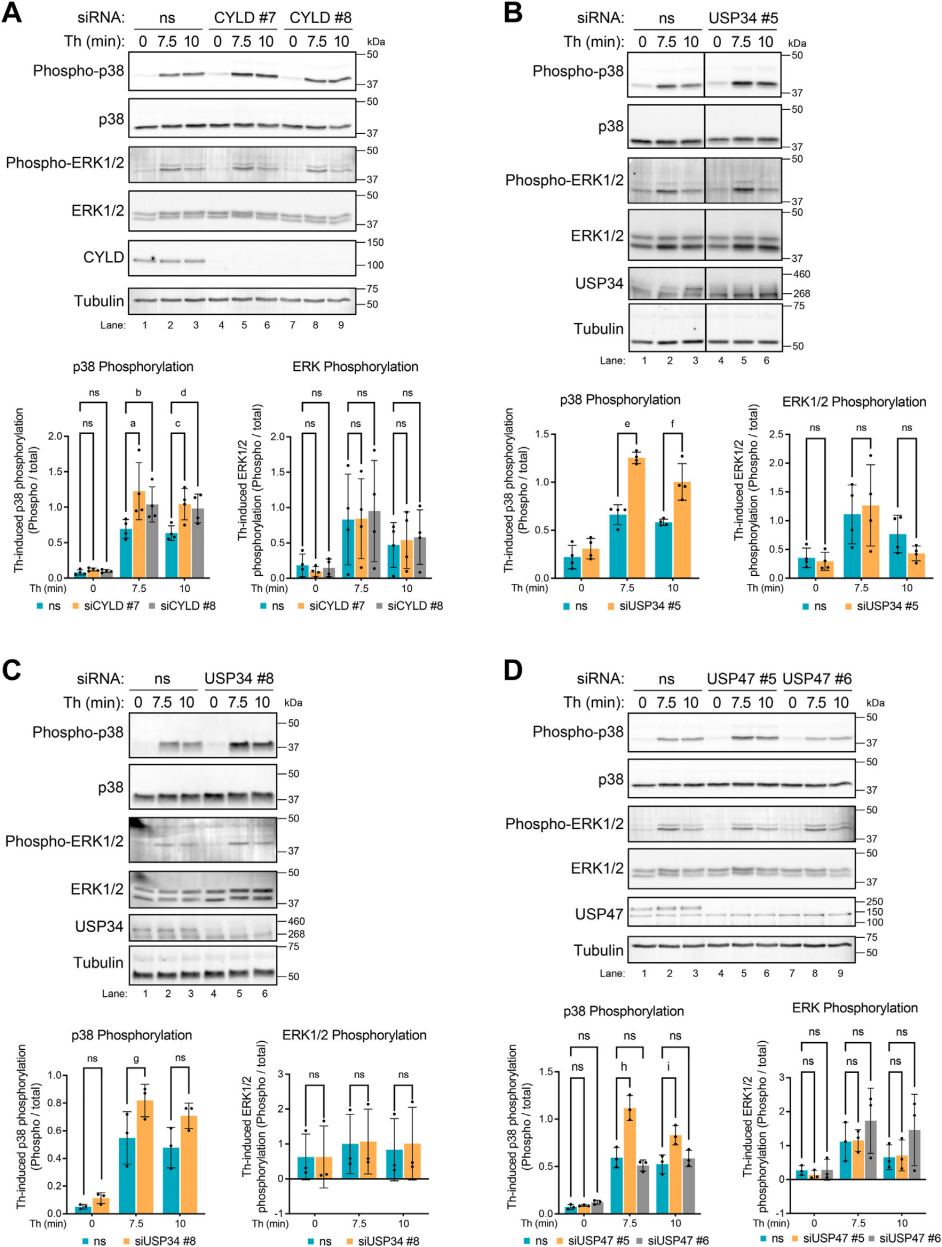
**Depletion of CYLD or USP34 using multiple individual siRNAs enhance thrombin-stimulated p38 phosphorylation but not ERK1/2 phosphorylation.***A*, HeLa cells transfected with CYLD or nonspecific (ns) siRNAs were incubated with thrombin (Th) for various times, lysed, and equivalent amounts of lysates were immunoblotted as indicated. HeLa cells were transfected with (*B*) USP34 or ns siRNA or (*C*) USP47 or ns siRNA, or (*D*) USP47 or ns siRNA treated with thrombin and immunoblotted as described above. The data (mean ± S.D.) for CYLD siRNA #7 (n=4), CYLD siRNA #8 (n=4), USP34 siRNA #5 (n=4), USP34 siRNA #8 (n=3), USP47 siRNA #5 (n=3), and USP47 siRNA #6 (n=3) are expressed as the change in phosphorylated p38 or ERK1/2 normalized to total p38 or ERK1/2 protein detected. Data were analyzed by two-way ANOVA, followed by Šídák's post hoc test. P values: a=0.0013, b=0.0378, c=0.0126, d=0.0365, e<0.0001, f=0.0002, g=0.0445, h<0.0001, i=0.0005; ns, not significant. See Supporting Information for full results of ANOVA.

To ensure the responses observed with the individual DUB siRNAs are specific and not due to off-target effects, we examined the effect of two individual siRNAs targeting CYLD, USP34, and USP47 on thrombin-stimulated p38 and ERK1/2 phosphorylation. In nonspecific siRNA transfected HeLa cells, thrombin induced a marked increase in p38 phosphorylation (Fig. 6*A*, lanes 1–3), which was significantly enhanced in cells depleted of CYLD expression using siRNA #7 or #8 (Fig. 6*A* lanes 4–9). In contrast, thrombin stimulated a marked increase in ERK1,2 phosphorylation at 7.5 min in nonspecific siRNA transfected cells (Fig. 6*A* lanes 1–3) that was comparable in CYLD-deficient cells (Fig. 6*A* lanes 1–9). Thrombin-promoted a significant increase in p38 phosphorylation in cells depleted of USP34 using siRNA #5 but had no effect on ERK1/2 phosphorylation compared to nonspecific siRNA transfected cells (Fig. 6*B* lanes 1–6). A similar effect was observed with USP34 siRNA #8 compared to nonspecific siRNA control transfected cells (Fig. 6*C* lanes 1–6). Unlike CYLD and USP34, only USP47 siRNA #5 significantly enhanced thrombin-stimulated p38 phosphorylation without effecting the ERK1/2 phosphorylation response compared to nonspecific siRNA transfected control cells (Fig. 6*D* lanes 1–6), whereas USP47 siRNA #6 failed to alter the thrombin-p38 response (Fig. 6*D* lanes 1–3 and 7–9). Moreover, USP47 siRNA #6 and not siRNA #5 appears to modulate thrombin-induced p38 signaling in endothelial cells (Fig. 5*C* lanes 1–12), opposite of that observed in HeLa cells. Given the potential off-target effects of USP47 SMARTpool siRNAs, the function of USP47 in thrombin-promoted p38–mediated responses was not further evaluated. Whereas two different individual siRNAs targeting CYLD or USP34 enhanced thrombin-p38 phosphorylation without altering ERK1/2 phosphorylation, suggesting that the siRNA effects are specific.

### CYLD and not UPS34 preferentially regulates thrombin-stimulated endothelial barrier permeability

**Figure 7:**
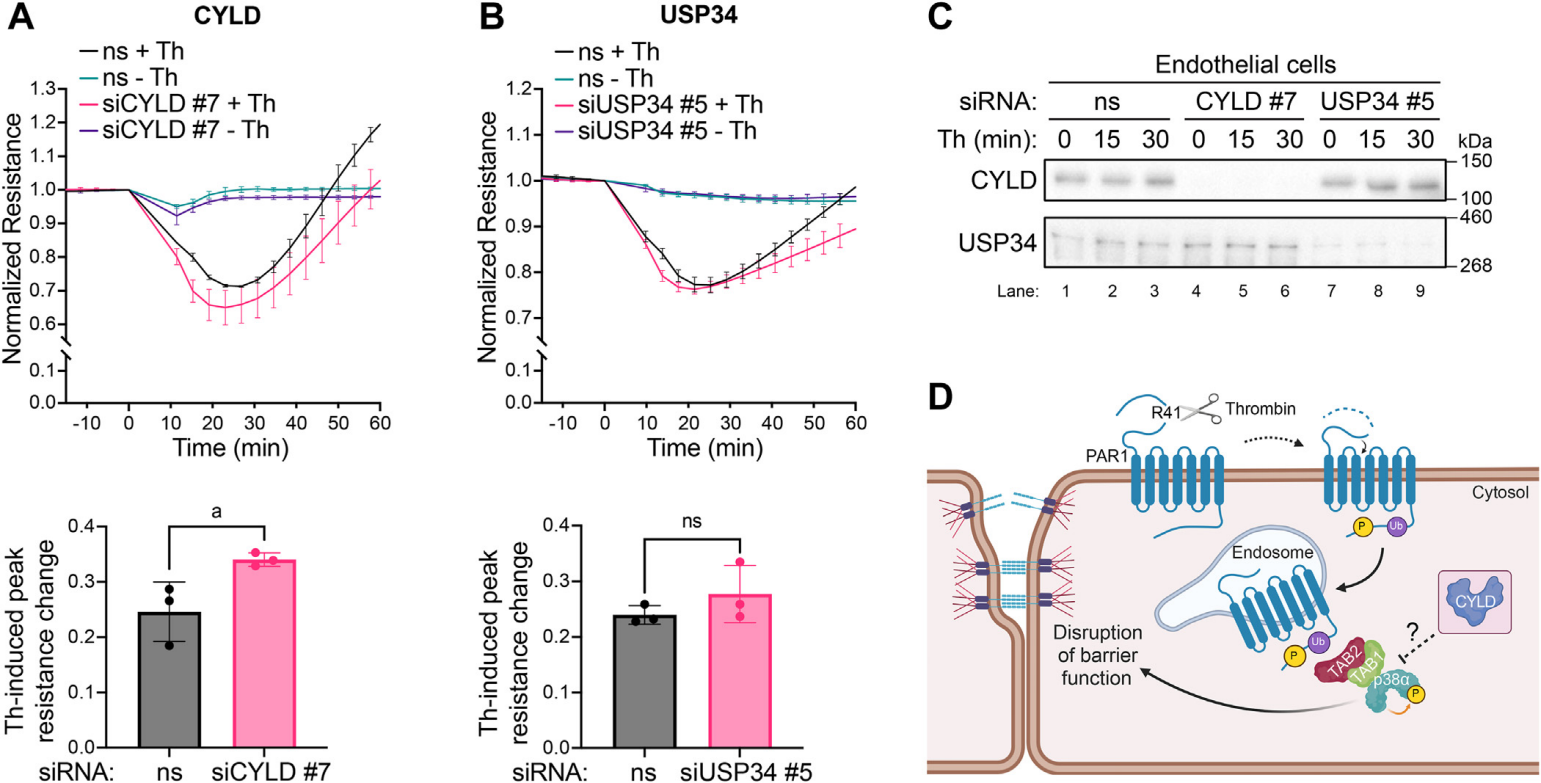
**Depletion of CYLD but not USP34 enhances thrombin-induced endothelial barrier permeability.** Endothelial EA.hy926 cells transfected with nonspecific (ns), CYLD- (*A*), and USP34- (*B*) specific siRNA were stimulated with or without thrombin (Th), and endothelial barrier permeability (resistance) was measured by ECIS. Changes in endothelial barrier resistance were normalized to baseline for each condition, and data expressed relative to maximum thrombin-induced change in resistance quantified from three independent experiments for CYLD and USP34 siRNA knockdown. The line graphs are from a representative experiment (mean ±S.D., performed in triplicate, n=3) for each DUB knockdown. The bar graphs are the peak change in resistance (mean ± S.D.) from the three independent experiments (n=3) and were analyzed by two-tailed student’s t test. P-value: a=0.0415; ns, not significant. *C*, cell lysates were prepared from the nonspecific, CYLD and USP34 siRNA transfected cells and immunoblotted as indicated. *D*, cartoon illustration of thrombin-activated PAR1 ubiquitin-driven p38 mediated endothelial barrier disruption regulation by CYLD.

To determine impact of CYLD and USP34 on thrombin-mediated inflammatory responses, endothelial cells were transfected with validated CYLD #7 and USP34 #5 siRNA and endothelial barrier disruption was examined using electric cell-substrate impedance sensing (ECIS). ECIS measures the dynamics of endothelial barrier function in living cells, the maximum peak change in impedance (resistance) and barrier recovery. Endothelial EA.hy926 cells form cell–cell junctions that maintain barrier function (38). In these studies, endothelial cells were transfected with CYLD siRNA #7 or USP34 siRNA #5 verified above (Figs. 5 and 6) and nonspecific control siRNA. In control siRNA transfected cells, thrombin induced a rapid and significant 25% reduction in resistance (barrier function) that peaked at 20 min, followed by return to baseline by 60 min. In contrast, thrombin caused a significantly greater 34% reduction in peak maximum resistance in endothelial cells deficient in CYLD expression compared to control siRNA transfected thrombin-stimulated cells (Fig. 7, *A* and *C* lanes 1–6), consistent with enhanced p38 signaling that we previously reported to promote thrombin-induced barrier disruption (10). However, depletion of USP34 with siRNA #5 had no significant effect on thrombin-induced endothelial barrier disruption compared to nonspecific siRNA-treated control cells (Fig. 7, *B* and *C* lanes 1–3 and 7–12), despite altering thrombin-stimulated p38 signaling (Figs. 5*B*, and 6B). These findings suggest that distinct DUBs differentially regulate thrombin-stimulated inflammatory responses (Fig. 7*D*).

### CYLD and USP34 differentially regulate thrombin-induced IL-6 cytokine production

**Figure 8:**
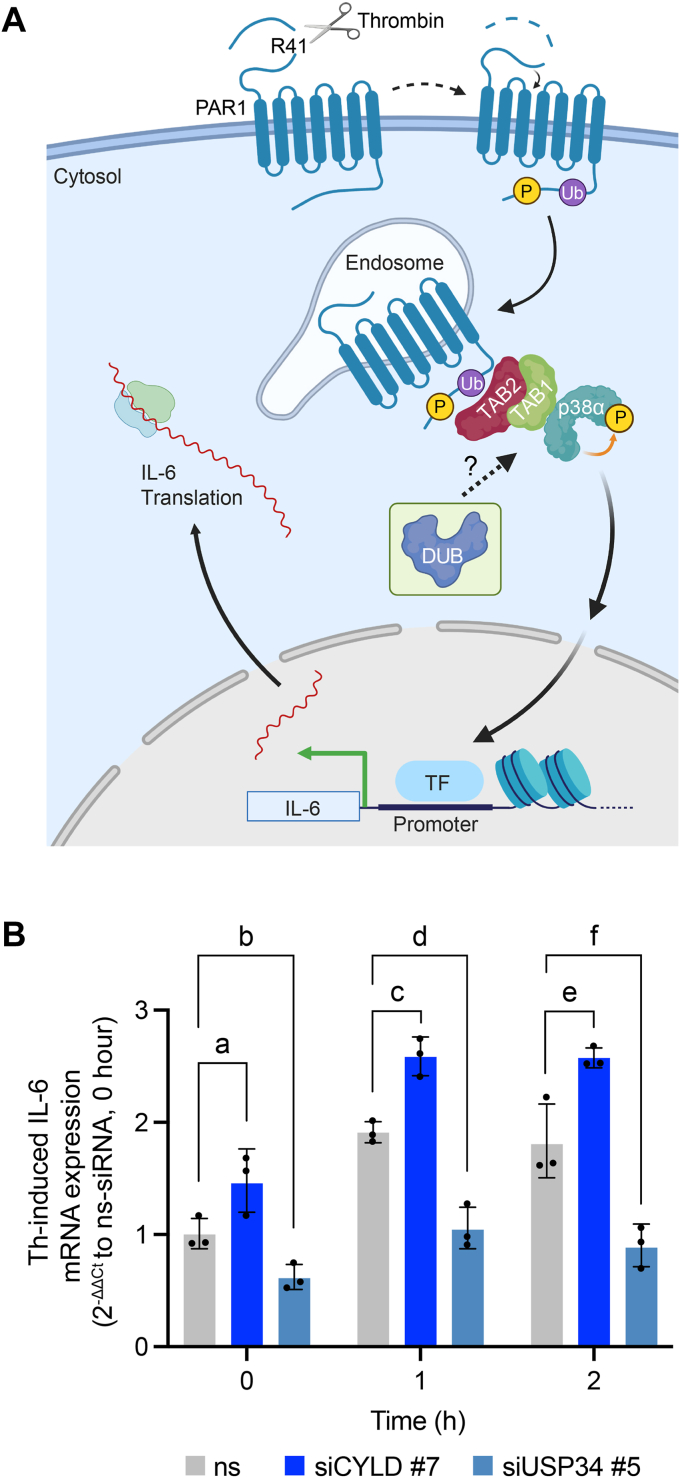
**Thrombin-induced IL-6 expression is differentially regulated by CYLD and USP34**. *A*, cartoon illustration of thrombin-activated PAR1-stimulated TAB1/TAB2-p38 mediated induction of interleukin-6 (IL-6) expression and potential regulation by deubiquitinating enzymes (DUBs). *B*, endothelial EA.hy926 cells were transfected with nonspecific (ns), CYLD, or USP34 specific siRNAs. Cells were then stimulated with or without thrombin (Th) for the indicated times. The effect of CYLD and USP34 siRNA-mediated depletion compared to nonspecific (ns) siRNA on thrombin-stimulated IL-6 expression was then quantified by RT-qPCR. Data (mean ± S.D., n = 3) normalized to 18S rRNA are expressed as 2^-ΔΔCt^ relative to ns siRNA and analyzed by two-way ANOVA, followed by Dunnett’s post hoc test. *p* values: a = 0.0132, b = 0.0017, c = 0.0455, d = 0.0002, e = 0.0189, and f < 0.0001. See Supporting information for full results of ANOVA. CYLD, cylindromatosis; PAR1, protease-activated receptor-1; RT-qPCR, quantitative real-time reverse transcription PCR; TAB, transforming growth factor-β–activated kinase-1–binding protein; USP, ubiquitin-specific protease.

Several endothelial GPCRs including PAR1 induce production of interleukin-6 (IL-6), a potent inflammatory cytokine (39, 40), which occurs through noncanonical TAB1-TAB2 activation of p38 signaling (13) (Fig. 8*A*). To determine the function of DUBs in thrombin-stimulated IL-6 production, endothelial cells were transfected with CYLD siRNA #7, USP34 siRNA #5, or nonspecific siRNA and IL-6 gene induction was measured quantitatively by RT-qPCR. In nonspecific siRNA transfected cells, a marked increase in IL-6 mRNA abundance was detected, following 1 to 2 h of thrombin stimulation (Fig. 8*B*). In CYLD-depleted cells (Fig. 8*B*), basal levels of IL-6 mRNA were significantly greater than nonspecific siRNA transfected control cells (Fig. 8*B*). However, despite higher basal IL-6 mRNA, thrombin induced a significant increase in IL-6 mRNA production in CYLD depleted cells than nonspecific siRNA control cells (Fig. 8*B*). In contrast to CYLD, the basal levels of IL-6 mRNA were significantly lower in cells depleted of USP34 expression (Figs. 7*C* and 8B) than nonspecific siRNA transfected cells (Fig. 8*B*). Nonetheless, thrombin caused a modest increase in IL-6 mRNA abundance in USP34 knockdown cells compared to untreated control cells (Fig. 8*B*). These data suggest that CYLD functions to attenuate thrombin-induced IL-6 production, whereas USP34 is important for promoting thrombin-stimulated IL-6 expression.

**Figure 9:**
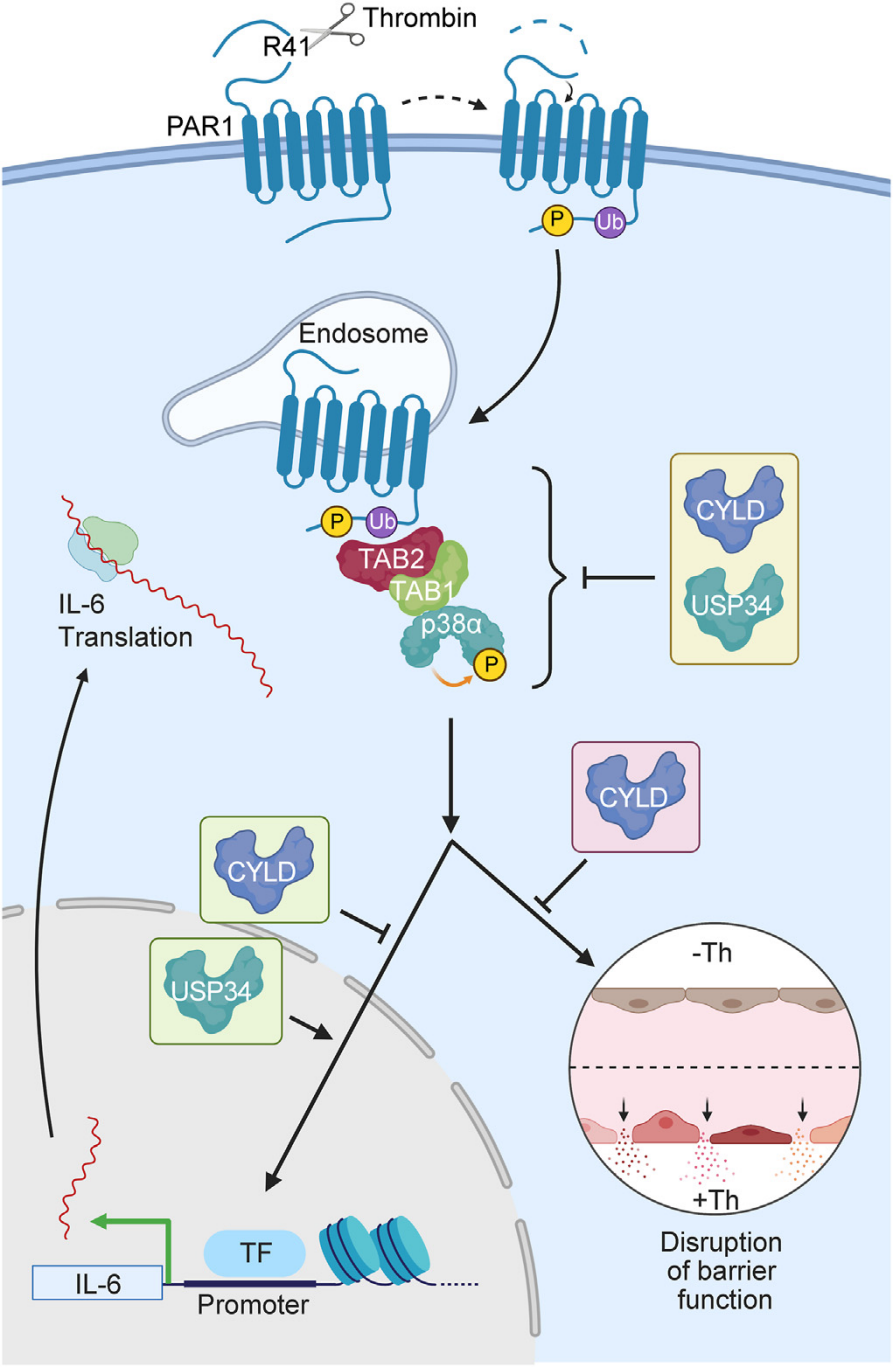
**Model of thrombin-activated PAR1 ubiquitin-driven p38-mediated inflammatory signaling regulation by CYLD *versus* USP34.** Thrombin activation of PAR1 promotes ubiquitin-dependent TAB1-TAB2 stimulated p38 MAPK signaling from endosomes. Both CYLD and USP34 regulate thrombin-activated PAR1-induced p38 signaling. However, CYLD functions to control thrombin-induced endothelial barrier permeability, whereas both CYLD and USP34 regulate thrombin-stimulated IL-6 mRNA expression. The precise mechanisms by which CYLD and USP34 function to control GPCR ubiquitin-driven p38 signaling are not known. CYLD, cylindromatosis; GPCR, G protein–coupled receptor; IL-6, interleukin-6; PAR1, protease-activated receptor-1; TAB, transforming growth factor-β–activated kinase-1–binding protein; USP, ubiquitin-specific protease.

CYLD and USP34 regulate GPCR-stimulated p38 signaling, however, the different DUBs appear to function in unique pathways to control GPCR-induced inflammatory responses. CYLD attenuates thrombin-p38–dependent stimulated barrier function and IL-6 production, whereas USP34 promotes IL-6 mRNA expression but does not alter endothelial barrier maintenance (Fig. 9). Although thrombin-activated PAR1 ubiquitination drives p38 activation *via* the TAB1–TAB2 complex, it is currently not know if CYLD or USP34 directly regulates PAR1, TAB1, TAB2, or p38 to impact thrombin-induced p38 signaling. These studies are the first to identify a subset of DUBs using a comprehensive DUB siRNA library screen that specifically regulate GPCR-p38 signaling and distinctly regulate endothelial inflammatory responses (Fig. 9).

## Discussion

The role and regulation of GPCR function by ubiquitination remains poorly understood. Most studies have linked ubiquitination to GPCR lysosomal or proteasomal degradation (3). However, ubiquitin can also directly modulate GPCR signaling. This has been well characterized for the GPCR PAR1, where thrombin triggers ubiquitin-dependent recruitment of TAB2-TAB1 and activation of p38 MAPK signaling on endosomes to promote endothelial inflammatory responses (10, 13, 14). Ubiquitination is a reversible process and deubiquitination is important for governing proper ubiquitin-dependent cellular responses. To identify DUBs that regulate thrombin-stimulated p38 signaling in human cultured endothelial cells, a SMARTpool siRNA library screen targeting 96 human DUBs was conducted. A similar siRNA library screen was performed in HeLa cells expressing PAR1. The dual screens identified nine DUBs including USP18, BAP1, CYLD, USP34, OTUD7A, UBTD2, USP51, USP 47, and JOSD1. We validated the function of two DUBs, CYLD, and USP34 that altered thrombin-stimulated p38 signaling in both cell types. Our study indicates that CYLD functions as a key regulator of thrombin-induced p38 activation and controls the magnitude of thrombin-induced endothelial barrier permeability and IL-6 cytokine expression. Whereas USP34 knockdown enhanced thrombin-promoted p38 signaling and reduced IL-6 cytokine expression but did not alter endothelial barrier integrity. In contrast to CYLD depletion, USP34 knockdown reduced basal IL-6 expression and thrombin-stimulated IL-6 expression. This study reveals the diversity of DUB function in regulating GPCR signaling and further suggests that specific DUBs distinctly regulate GPCR-p38–mediated inflammatory responses.

In this study an unbiased siRNA library screen approach was used to identify the relevant DUBs that control thrombin-stimulated p38 signaling in two cell types. Other studies used different approaches to identify DUBs that regulate activated GPCR ubiquitination and lysosomal degradation. USP33 was identified in a yeast two-hybrid screen with β-arrestin (41). USP33 and its homolog USP20 were shown to decrease β2-adrenergic receptor ubiquitination, which reduced receptor lysosomal degradation, and enhanced recycling and cellular resensitization (42). USP14 was identified in a proteomics screen and shown to decrease ubiquitination of agonist activated CXCR4 chemokine receptor and lysosomal degradation (43, 44). USP8 was identified in a far Western cDNA library screen using signal-transducing adaptor molecule (STAM) (45) and appears to regulate CXCR4 lysosomal degradation indirectly *via* modulation of hepatocyte growth factor–regulated tyrosine kinase substrate (46). STAM and hepatocyte growth factor–regulated tyrosine kinase substrate are components of the endosomal sorting complex required for transport-0 that recognize and initiate ubiquitinated cargo sorting within the endosomal–lysosomal pathway. Thus, multiple strategies have been used to identify DUBs that regulate GPCR function. However, a DUB siRNA library screen conducted in two different cell lines with endogenous or ectopically expressed receptor is a powerful approach to identify new and relevant DUBs that regulate GPCR-mediated cellular responses.

The regulation of DUB activity is important for proper cellular responses. DUB activity is controlled through relative abundance, protein–protein interactions, subcellular localization, and posttranslational modifications (26, 27). DUB phosphorylation impacts function as demonstrated for CYLD regulation of tumor necrosis factor receptor–associated factors 2 (TRAF2) and 6 (TRAF6) proteins (47, 48). Phosphorylation of CYLD at S418 by NF-κB signaling impairs CYLD catalytic activity enhancing TRAF2 and TRAF6 degradation (29, 30). In addition, CYLD can modulate NF-κB signaling (49). Although thrombin activates NF-κB signaling in endothelial cells (50, 51), it is not known if thrombin regulates CYLD function *via* phosphorylation. Moreover, CYLD has been implicated in thrombin-induced endothelial barrier permeability through a pathway mediated by MALT1 cleavage of CYLD that results in microtubule destabilization (52). Currently, it is not known whether the impact of CYLD on p38 signaling and control of endothelial barrier integrity is independent of MALT1 function. In contrast to CYLD, the function of USP34 phosphorylation sites is not known. CYLD is also ubiquitinated by the Skp1-Cullin1-F-box protein β-transducin repeat-containing protein, which promotes proteasomal degradation (53). A ubiquitination site has also been identified for USP34 (Fig. 3), however the role of ubiquitination in regulating USP34 function has yet to be determined.

In addition to posttranslational modifications, DUB function is regulated by subcellular localization which is often mediated by protein–protein interactions with components of large complexes such as the proteosome. Several DUBs are known to bind to the proteasome and function to deubiquitinate proteins that are destined for degradation, a process that facilitates recycling of ubiquitin (54, 55, 56). A systematic analysis of the subcellular localization of 66 different GFP-tagged DUBs including CYLD but not UPS34 or USP47 was conducted in HeLa cells (57). In this study, GFP-tagged CYLD was shown to reside in the cytoplasm and nucleus, consistent with endogenous CYLD subcellular localization as shown in Figure 4*B*. However, other studies have reported endogenous and ectopically expressed CYLD localization to microtubules (58, 59), which was not obvious in our HeLa cell studies. Similar to CYLD, subcellular localization of USP34 was detected in the cytoplasm and nucleus and is consistent with its known function to regulate the cytoplasmic protein Axin and DNA damage repair (35, 36). These findings raise the intriguingly possibility that CYLD and USP34 differentially modulate thrombin-induced p38 activity in distinct subcellular compartments, including endosomes and the nucleus to promote differential regulation of endothelial barrier permeability *versus* IL-6 production.

The role of ubiquitin in driving GPCR signaling is an emerging concept. Several GPCRs including mGluR7, CXCR2, and parathyroid hormone receptor promote ubiquitin-dependent signaling *via* recruitment of β-arrestin to the plasma membrane (12, 60, 61). The CXCR4 receptor also drives ubiquitin-dependent signaling indirectly via modulation of STAM ubiquitination (11). GPCR ubiquitination is also influenced by biased agonists as demonstrated for the μ-opioid receptor (62). Interestingly, biased ligands induce ubiquitination of the β-adrenoreceptor *via* distinct E3 ubiquitin ligases (63, 64, 65). We showed that thrombin induces PAR1 ubiquitination and recruitment of TAB2-TAB1 to endosomes, which promotes noncanonical p38 signaling and endothelial barrier disruption (10, 14). Thrombin-stimulated p38 signaling does not integrate with the RhoA/myosin light chain pathway to control endothelial barrier permeability (20). In a recent study, we sought to determine how thrombin-stimulated endosomal p38 signaling contributes to endothelial barrier disruption using quantitative mass spectrometry and identified a rich array of candidate proteins including ERK1/2 and α-catenin that may regulate p38-dependent barrier disruption independent of RhoA/myosin light chain (24). Other subsets of GPCRs including P2Y1 receptor, histamine H1 and H2 and prostaglandin receptors also stimulate noncanonical ubiquitin- and TAB2-TAB1–dependent p38 activation (13). Currently, it is not known whether CYLD and/or USP34 directly modulates ubiquitination of the GPCR PAR1 and/or other p38 pathway components such as p38, TAB1, TAB2, NEDD4-2, and c-Src to control noncanonical p38 MAPK signaling. In fact, several GPCR ubiquitin–driven p38 pathway components are known to be ubiquitinated including TAB1 (66), NEDD4-2 (14, 67), and c-Src (68), but it is unclear if any of the pathway components are direct targets of CYLD and/or USP34.

Our study also provides critical insight into the function of CYLD and USP34 in regulating GPCR-p38 signaling and cytokine IL-6 production. We found that depletion of CYLD enhanced basal IL-6 production and amplified thrombin-induced IL-6 production (Fig. 8*B*), which might be attributed to the increased basal p38 phosphorylation observed in CYLD knockdown at the 0 min time point (Fig. 5*A*). The mechanism responsible for enhanced p38 basal signaling in CYLD depleted cells is not known. In contrast, USP34 knockdown diminished basal IL-6 expression and decreased thrombin-induced IL-6 production (Fig. 8*B*), even though both CYLD and USP34 enhance thrombin-p38 signaling. As mentioned above, subcellular localization of the DUB and impact on compartmentalized p38 signaling may contribute to differential regulation of thrombin-induced IL-6 production. Clearly, additional studies are needed to interrogate the mechanisms by which CYLD and USP34 differentially modulate thrombin-induced endothelial barrier disruption and IL-6 production.

## Experimental procedures

### Reagents and antibodies

Human α-Thrombin was obtained from Enzyme Research Laboratories (#HT1002a). The following antibodies were obtained from Cell Signaling Technology p38 MAPK (#9212), phospho-p38 MAPK (#4511), p44/42 MAPK ERK1/2 (#9102), phospho-p44/42 MAPK ERK1/2 (#9106), and CYLD (#8462). Other antibodies included USP47 (#ab72143, Abcam), USP34 (#A300-824A, Bethyl Laboratories). The p38, CYLD, USP34, and USP47 antibodies were validated by siRNA as shown in Figs. 1*B*, and 5, *A*–*C*, respectively. The ERK1/antibody #9102 was validated by siRNA as shown on the CST website. Horseradish peroxidase-conjugated goat-anti rabbit (#1706515), horseradish peroxidase-conjugated goat-anti mouse (#1706516), StarBright Blue 520 goat anti-mouse (#12005867), StarBright Blue 700 goat anti-rabbit (#12004162), and hFAB Rhodamine Tubulin (#12004166) were from Bio-Rad. Vinculin antibody (#V9131) and SB203580 (#S8307) were from Sigma-Aldrich.

### Cell culture

HUVEC-derived EA.hy926 cells were obtained from American Type Culture Collection (ATCC), grown and propagated, and used between P2 and P10 according to ATCC instructions and as described (22, 24). HUVEC-derived EA.hy926 cells are authenticated by short tandem repeat profiling as noted by ATCC. HeLa cells stably expressing FLAG-tagged PAR1 WT cloned in pBJ were generated and cultured as previously described (23, 69). All cell lines are routinely monitored for *mycoplasma* using Venor GeM *Mycoplasma* detection kit (Sigma-Aldrich, #MP0025-1KT) per the manufacturer’s instructions.

### DUB siRNA library screen

Human ON-TARGETplus siRNA library targeting 96 DUBs genes consisting of SMARTpool siRNA (four individual siRNAs targeting each gene) in a 96-well format was purchased from Horizon Discovery Ltd (#G-104705-05). Endothelial EA.hy926 cells were seeded at 0.7 × 10^5^ cells per well in 48-well plates and PAR1 HeLa cells were seeded at 7 × 10^3^ cells per well in 96-well plates and grown overnight. Endothelial cells were transfected with 96 distinct DUB SMARTpool siRNAs at 28 nM using TransIT-X2 Dynamic Delivery System, and PAR1 HeLa cells were transfected with the same DUB SMARTpool siRNAs at 50 nM using Oligofectamine Transfection Reagent according to the manufacturer’s instructions. Endothelial and HeLa cells were also transfected with 50 nM p38α siRNA, 25 nM of PAR1-208 siRNA, and 50 nM nonspecific siRNA as controls. After 72 h of transfection, cells were serum-starved and then stimulated with 10 nM thrombin for 7.5 min (endothelial cells) or 5 min (PAR1 HeLa cells) at 37 °C or left untreated 0 min. Cells were also pretreated with 5 μM SB203580 or dimethylsulfoxide for 30 min before thrombin stimulation as a control. After stimulation, cells were placed on ice, washed and lysed in 2 ×Laemmli Sample Buffer supplemented with 200 mM DTT. Cell lysates were resolved by SDS-PAGE, transferred to polyvinylidene fluoride membrane, and immunoblotted with antibodies as indicated. Immunoblots were developed and imaged using ChemiDoc Imaging System (Bio-Rad) with appropriate filter set for StarBright 520, StarBright 700, and chemiluminescence. Signal was quantified by densitometry with NIH ImageJ software (https://imagej.net/ij/, 1997–2018) or Image Lab 6.1 (Bio-Rad; http://www.bio-rad.com/en-us/sku/1709690-image-lab-software) and displayed as fold increase relative to nonspecific siRNA control.

### Individual siRNAs

AllStars negative control nonspecific siRNA (#1027281) and p38α-specific siRNA 5′-AACTGCGGTTACTTAAACATA-3′, and custom designed PAR1-208 siRNA 5′-AGAUUAGUCUCCAUCAAUA-3′ (70) were purchased from Qiagen. The following individual DUB siRNAs were used in validation studies including: CYLD 5′-GGACAUGGAUAACCCUAUU-3’ (#J-004609-05), 5′-AGAGAUAUCUACAGACUUU-3’ (#J-004609-06), 5′- GGAGAGUACUUGAAGAUGU-3 (#J-004609-07), 5′- GAAGGUUGGAGAAACAAUA-3’ (#J-004609-08), and UPS34 5′- GCAGGGAAGUUCUGACGAA-3’ (#J-006082-05), 5′- AACAGAUCAGUAGUAAUU-3’ (#J-006082-06), 5′- GCAGCUAUCCAGUAUAUUA-3’ (#J-006082-07), 5′- CCAUGUGACUGGAGAUUUA-3’ (#J-006082-08) and USP47 5′- GCAACGAUUUCUCCAAUGA-3 (#J-006093-05), 5′- CAACAUGUCAGCAGGAUAA-3’ (#J-006093-06), 5′- GCUGUCGCCUUGUUAAAUA-3’ (#J-006093-07), 5′- CGCAAUACAUGCAAGAUAA-3’ (3 J-006093-08).

### Quantitative real-time reverse transcription PCR

RNA was isolated from endothelial EA.hy926 cells seeded at 9.6 × 10^5^ cells per well in a 6-well plate and PAR1 HeLa cells seeded at 6 × 10^5^ cells per well of a 6-well plate grown overnight using Direct-zol RNA Miniprep Plus Kit (#R2072, Zymo Research). Total RNA was quantified and 1 μg total RNA was utilized for cDNA synthesis reaction with the SuperScript IV VILO Master Mix with ezDNase enzyme kit (#111766050, Thermo Fisher Scientific). RT-qPCR was performed with TaqMan Fast Advanced Master Mix (#4444964, Thermo Fisher Scientific) and TaqMan Gene Expression Probes CYLD (#Hs01031576_m1), USP34 (#Hs00611330_m1), USP47 (#Hs00215725_m1), USP51 (#Hs01651394_s1), IL-6 (#Hs00174131_m1), and 18S (#Hs03003631_g1, primer-limited) using a QuantStudio three Real-Time PCR System (Thermo Fisher Scientific). DUB and IL-6 mRNA transcript levels were analyzed using the comparative CT (threshold cycle) method, normalized to 18S ribosomal RNA expression. Briefly, the number of cycles until threshold (Ct) was determined for each target, and the Ct value for 18S was subtracted from the Ct value for each target, the differences in expression was then determined using the 2^-ΔΔCt^ method.

### Signaling assays and immunoblotting

Signaling assays were performed essentially as described (10, 13, 14). Cells were serum-starved and pretreated with 5 μM SB203580 or dimethylsulfoxide vehicle control for 30 min at 37 °C. Cells were then stimulated with 10 nM thrombin for various times. Cells were lysed, protein concentrations determined using a bicinchoninic acid protein assay (Thermo Fisher Scientific) and equivalent amounts of cell lysates were resolved by SDS-PAGE, transferred to polyvinylidene fluoride membranes, and immunoblotted with antibodies as indicated. Membranes were developed by chemiluminescence or fluorescence detection using a ChemiDoc Imaging System (Bio-Rad), and quantified, analyzed by densitometry using with NIH ImageJ software or Image Lab 6.1 (Bio-Rad).

### Immunofluorescence confocal microscopy

HeLa cells stably expressing FLAG-tagged PAR1 WT were plated at 3 × 10^4^ cells per well in a 12-well plate and grown on fibronectin-coated coverslips for 24 h. Cells were fixed and permeabilized with methanol at −20 °C for 10 min and blocked with 2% bovine serum albumin/PBS for 60 min at room temperature. Primary antibodies rabbit anti-CYLD (#PA5-29795, Invitrogen) at 1:200 dilution, rabbit anti-USP34 (#A300-824A, Bethyl Laboratories) at 1:200 dilution, and rabbit anti-USP47 (#ab72143, Abcam) at 1:500 dilution were used for staining. Secondary antibody goat anti-Rabbit IgG (H + L) conjugated to Alexa Fluor 488 (A-11008, Invitrogen) at 1:2000 dilution was used. All the antibodies were prepared in the blocking buffer and were incubated with coverslips for 60 min. Nuclei were stained with Hoechst 33342 solution prepared in blocking buffer. Coverslips were mounted with ProLong Gold Antifade Mountant (#P36934, Invitrogen).

Confocal microscopy image acquisition was perform using Olympus IX81 spinning-disk Microscope, equipped with a CoolSNAP HQ2 CCD Camera (Andor), a 60 × 1.40 NA oil PlanApo objective lens (Olympus), and appropriate filters. At least three random fields of view for each coverslip were acquired using MetaMorph software (MDS Analytical Technologies; http://www.moleculardevices.com/Products/Software/Meta-Imaging-Series/MetaMorph.html) with multiple focal positions. Image stacks were processed to generate Z-projection (maximum-intensity method) images using NIH Fiji software (https://imagej.net/ij/, 1997–2018).

### Electrical cell impedance sensing

Endothelial EA.hy926 cells were seeded onto cysteine- and collagen-coated gold microwell 8W10E+ ECIS array (Applied Biophysics). Cells were transfected with 28 nM nonspecific, CYLD, USP34, and USP47 siRNA using TransIT X2 (Mirus Bio) according to the manufacturer’s instructions. Cells were then allowed to reach confluence for 48 to 72 h until impedance reached 3000 Ω. The baseline barrier was recorded and established by multiple frequency time. Changes in resistance, a measure of barrier function, was determined after the addition of thrombin and recording continued until the barrier recovered to the previously established baseline impedance as we described (20).

### Model and prediction analysis

Schematics and Figures were created in Adobe Illustrator and Photoshop. The cartoons were created with BioRender.com. UniProt Knowledgebase was used to query CYLD, UBP34 (USP34), and UBP47 (USP47) subcellular localization based on curated information (32).

### Statistical analysis

Data were analyzed using Prism 9.0 statistical software (GraphPad Software) and Microsoft Excel. Statistical analysis methods are indicated in the Figure legends.

## Data availability

All data are contained within the article or shown as supplemental data.

## Supporting information

This article contains supporting information.

## Conflict of interest

J. T. reports financial support was provided by 10.13039/100000002National Institutes of Health. The other authors declare that they have no conflicts of interest with the contents of this article.
