# Supplementary material for: An siRNA library screen identifies CYLD and USP34 as deubiquitinases that regulate GPCR-p38 MAPK signaling and distinct inflammatory responses
Source: J Biol Chem. 2023 Oct 20;299(12):105370. doi: 10.1016/j.jbc.2023.105370 (PMC10694601; doi:10.1016/j.jbc.2023.105370)
Supplement: Supporting Information [file mmc1.pdf]

Supporting Information

An siRNA library screen identifies CYLD and USP34 as deubiquitinases that regulate GPCR-p38 MAPK signaling and distinct inflammatory responses

Norton Cheng[1,2] and JoAnn Trejo[1*]
From the [1]Department of Pharmacology, School of Medicine, University of California, San Diego, La Jolla, CA 92093; [2]Biomedical Sciences Graduate Program, School of Medicine, University of California, San Diego, La Jolla, CA 92093

Supporting Information file includes:

1. **Supplemental Figure 1.**
HeLa cell DUB siRNA library screen immunoblots of thrombin-stimulated p38 phosphorylation.

2. **Supplemental Figure 2.**
Endothelial EA.hy926 cells DUB siRNA library screen immunoblots of thrombin-stimulated p38 phosphorylation.

3. **Supporting Dataset S1.**
HeLa cell DUB siRNA library screen quantified data

4. **Supporting Dataset S2.**
Endothelial EA.hy926 cell DUB siRNA library screen quantified data

5. **Supporting Dataset S3.**
Full results of ANOVA

Supplemental Figure 1

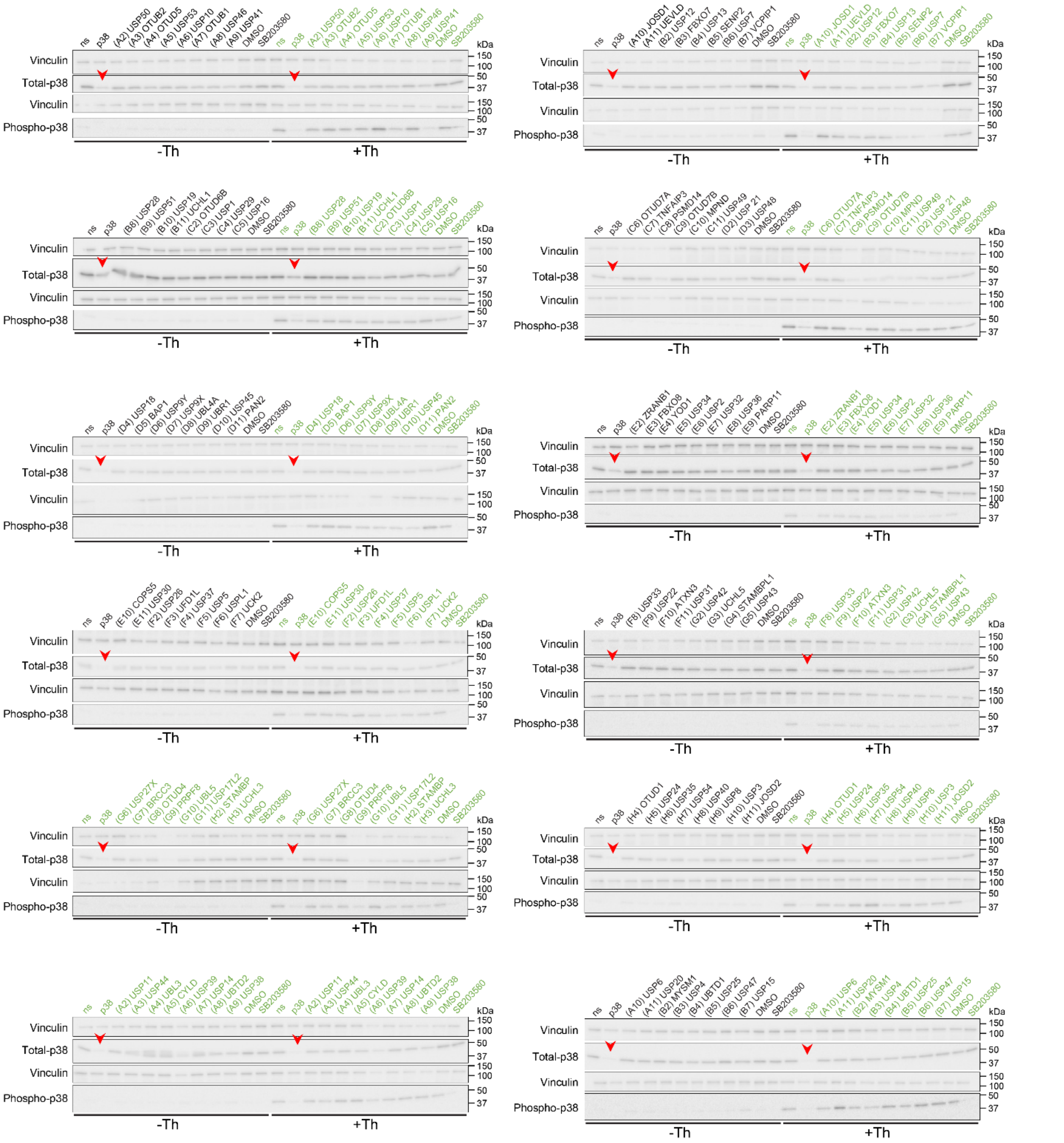

Supplemental Figure S1. DUB siRNA library screen of thrombin-induced p38 phosphorylation in PAR1-HeLa cells. Immunoblots of cell lysates from thrombin (Th) stimulated p38 phosphorylation assays conducted in PAR1-expressing HeLa cells transfected with non-specific (ns) siRNA, p38α siRNA or DUB siRNA SMART pools. Cell lysates from PAR1-HeLa cells pre-treated with the p38 inhibitor SB203580 or DMSO and then stimulated with thrombin were also

immunoblotted as indicated. Cell lysates were immunoblotted for vinculin as loading control. Red arrowhead indicates p38α depleted samples with and without thrombin treatment.

Supplemental Figure 2

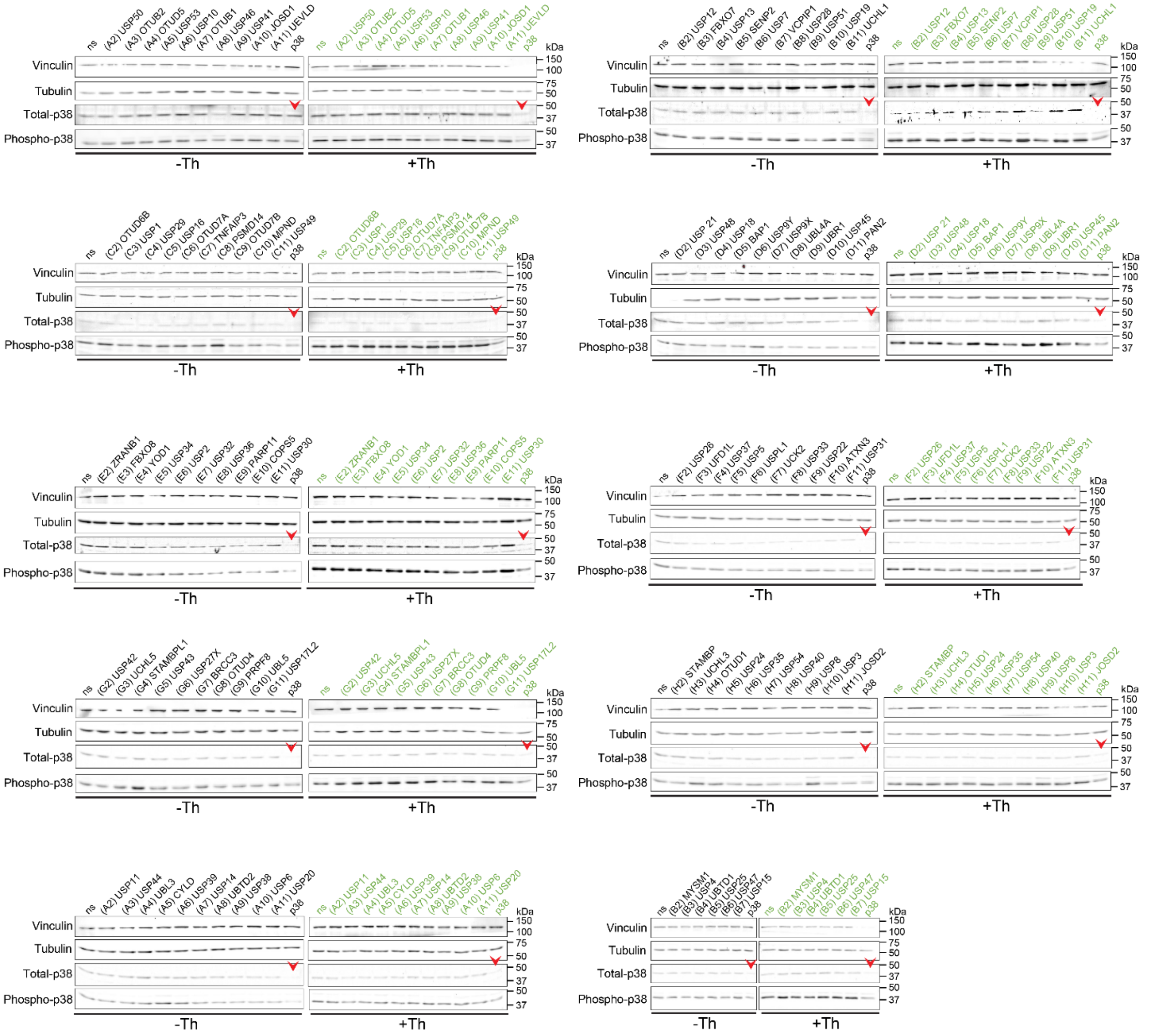

Supplemental Figure S2. DUB siRNA library screen of thrombin-induced p38 phosphorylation in endothelial EA.hy926 cells. Immunoblots of cell lysates from thrombin (Th) stimulated p38 phosphorylation assays conducted in endothelial cells expressing endogenous PAR1 transfected with non-specific (ns) siRNA, p38α siRNA or DUB siRNA SMART pools. Cell lysates were immunoblotted for vinculin and tubulin as loading controls. Red arrowhead indicates p38α depleted samples with and without thrombin treatment.

Dataset S3. Figure 6 and 8, full results of ANOVA

Figure 6

Panel A: CYLD-KD

p38

ANOVA table	SS	DF	MS	F (DFn, DFd)	P value
Interaction	0.2894	4	0.07234	F (4, 27) = 1.895	P=0.1402
Time	5.666	2	2.833	F (2, 27) = 74.22	P<0.0001
siRNA	0.6852	2	0.3426	F (2, 27) = 8.975	P=0.0010
Residual	1.031	27	0.03817		

ERK1/2

ANOVA table	SS	DF	MS	F (DFn, DFd)	P value
Interaction	0.03913	4	0.009782	F (4, 27) = 0.05178	P=0.9947
Time	3.224	2	1.612	F (2, 27) = 8.534	P=0.0013
siRNA	0.03437	2	0.01718	F (2, 27) = 0.09097	P=0.9133
Residual	5.101	27	0.1889		

Panel B: USP34-KD (siRNA #5)

p38

ANOVA table	SS	DF	MS	F (DFn, DFd)	P value
Interaction	0.5967	2	0.2983	F (2, 18) = 10.03	P=0.0012
Time	4.789	2	2.394	F (2, 18) = 80.53	P<0.0001
siRNA	1.844	1	1.844	F (1, 18) = 62.03	P<0.0001
Residual	0.5352	18	0.02973		

ERK1/2

ANOVA table	SS	DF	MS	F (DFn, DFd)	P value
Interaction	0.2441	2	0.1220	F (2, 18) = 0.7813	P=0.4727
Time	3.118	2	1.559	F (2, 18) = 9.980	P=0.0012
siRNA	0.03792	1	0.03792	F (1, 18) = 0.2427	P=0.6282
Residual	2.812	18	0.1562		

Panel C: USP34-KD (siRNA #8)

p38

ANOVA table	SS	DF	MS	F (DFn, DFd)	P value
Interaction	0.03650	2	0.01825	F (2, 12) = 1.344	P=0.2974
Time	1.264	2	0.6318	F (2, 12) = 46.51	P<0.0001
siRNA	0.1572	1	0.1572	F (1, 12) = 11.57	P=0.0053
Residual	0.1630	12	0.01358		

ERK1/2

ANOVA table	SS	DF	MS	F (DFn, DFd)	P value
Interaction	0.02243	2	0.01122	F (2, 12) = 0.01435	P=0.9858
Time	0.5278	2	0.2639	F (2, 12) = 0.3376	P=0.7201
siRNA	0.03009	1	0.03009	F (1, 12) = 0.03849	P=0.8477
Residual	9.382	12	0.7818		

Panel D: USP47-KD

p38

ANOVA table	SS	DF	MS	F (DFn, DFd)	P value
Interaction	0.3476	4	0.08689	F (4, 18) = 12.80	P<0.0001
Time	2.193	2	1.096	F (2, 18) = 161.6	P<0.0001
siRNA	0.4583	2	0.2291	F (2, 18) = 33.76	P<0.0001
Residual	0.1222	18	0.006787		

ERK1/2

ANOVA table	SS	DF	MS	F (DFn, DFd)	P value
Interaction	0.5444	4	0.1361	F (4, 18) = 0.4202	P=0.7920
Time	5.591	2	2.795	F (2, 18) = 8.630	P=0.0024
siRNA	1.409	2	0.7044	F (2, 18) = 2.175	P=0.1426
Residual	5.830	18	0.3239		

Figure 8

Panel B:

ANOVA table	SS	DF	MS	F (DFn, DFd)	P value
Interaction	0.1051	4	0.02627	F (4, 18) = 0.5511	P=0.7007
Time	3.793	2	1.896	F (2, 18) = 39.78	P<0.0001
siRNA	8.622	2	4.311	F (2, 18) = 90.43	P<0.0001
Residual	0.8581	18	0.04767		
